# 3,3′-(1,3,5,7-Tetra­oxo-2,3,6,7-tetra­hydro-1*H*,5*H*-pyrrolo[3,4-*f*]isoindole-2,6-di­yl)dipropanoic acid *N*,*N*-dimethyl­formamide disolvate

**DOI:** 10.1107/S1600536809045437

**Published:** 2009-11-04

**Authors:** Chun-Sheng Ling, Xu Wang, Yun Liu, Qiang Wu

**Affiliations:** a Institute of Pharmacy, Henan University, Kaifeng 475004, People’s Republic of China

## Abstract

In the title compound, C_16_H_12_N_2_O_8_·2C_3_H_7_NO, the complete tricyclic compound is generated by a crystallographic centre of symmetry. In the crystal, the tricycle is linked to two adjacent *N*,*N*-dimethyl­formamide solvent mol­ecules by O—H⋯O hydrogen bonds.

## Related literature

For a related structure and background, see: Wang & Wei (2005 ▶).
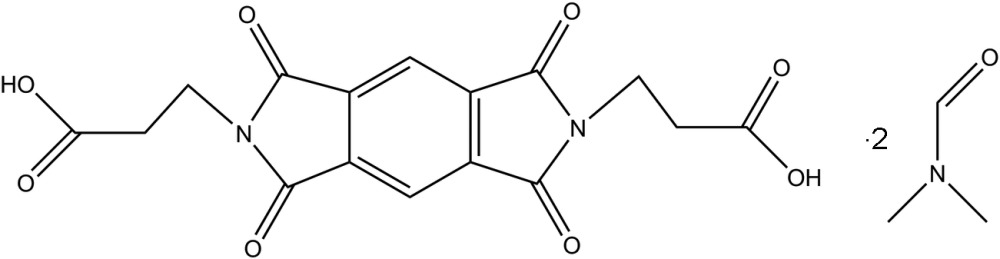


## Experimental

### 

#### Crystal data

C_16_H_12_N_2_O_8_·2C_3_H_7_NO
*M* *_r_* = 506.47
Monoclinic, 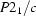

*a* = 12.542 (8) Å
*b* = 8.611 (6) Å
*c* = 12.902 (9) Å
β = 118.774 (8)°
*V* = 1221.3 (14) Å^3^
*Z* = 2
Mo *K*α radiation
μ = 0.11 mm^−1^
*T* = 296 K
0.33 × 0.31 × 0.10 mm

#### Data collection

Bruker SMART CCD diffractometer
Absorption correction: none
12363 measured reflections
2386 independent reflections
1745 reflections with *I* > 2σ(*I*)
*R*_int_ = 0.037

#### Refinement

*R*[*F* ^2^ > 2σ(*F* ^2^)] = 0.047
*wR*(*F* ^2^) = 0.133
*S* = 1.06
2386 reflections
167 parameters
15 restraints
H atoms treated by a mixture of independent and constrained refinement
Δρ_max_ = 0.44 e Å^−3^
Δρ_min_ = −0.30 e Å^−3^

### 

Data collection: *SMART* (Bruker, 2001 ▶); cell refinement: *SAINT-Plus* (Bruker, 2001 ▶); data reduction: *SAINT-Plus*; program(s) used to solve structure: *SHELXS97* (Sheldrick, 2008 ▶); program(s) used to refine structure: *SHELXL97* (Sheldrick, 2008 ▶); molecular graphics: *PLATON* (Spek, 2009 ▶); software used to prepare material for publication: *PLATON*.

## Supplementary Material

Crystal structure: contains datablocks global, I. DOI: 10.1107/S1600536809045437/hb5179sup1.cif

Structure factors: contains datablocks I. DOI: 10.1107/S1600536809045437/hb5179Isup2.hkl

Additional supplementary materials:  crystallographic information; 3D view; checkCIF report

## Figures and Tables

**Table 1:** Hydrogen-bond geometry (Å, °)

*D*—H⋯*A*	*D*—H	H⋯*A*	*D*⋯*A*	*D*—H⋯*A*
O2—H2⋯O5^i^	0.82	1.78	2.583 (3)	166

Symmetry code: (i) 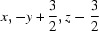.

**Fig. 1.**
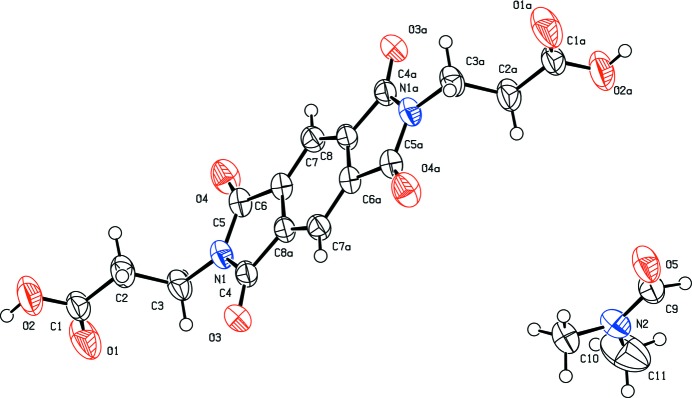
The molecular structure of (I), showing displacement ellipsoids drawn at the 50% probability level.

## supplementary crystallographic information

### Experimental

3-Bromopropanoic acid (2 mmol, 0.306 g) and pyrrolo[3,4-*f*]isoindole-1,3,5,7(2*H*,6H)-tetraone (1 mmol, 0.360 g) were dissolved in 20 ml of the mixed solvent of *N*,*N*-dimethylformamide and water in a ratio of 1:2 (v/v). The mixture was heated in a Teflon-lined steel autoclave inside a programmable electric furnace at 353 K for three days. After cooling the autoclave to room temperature, colourless slabs of (I) were obtained.

### Refinement

H9A aom was located by Fourier map, other H atoms were geometrically placed with C—H = 0.93–0.97 Å and O—H = 0.82 Å, and were refined as riding with *U*_iso_(*H*)=1.2*U*_eq_(C_methylene_ and C in phenyl ring) and *U*_iso_(H)=1.5*U*_eq_(O and C_methyl_).

### Crystal data

**Table:**

C_16_H_12_N_2_O_8_·2C_3_H_7_NO	*F*(000) = 532
*M**_r_* = 506.47	*D*_x_ = 1.377 Mg m^−^^3^
Monoclinic, *P*2_1_/*c*	Mo *K*α radiation, λ = 0.71073 Å
Hall symbol: -P 2ybc	Cell parameters from 2660 reflections
*a* = 12.542 (8) Å	θ = 3.0–23.7°
*b* = 8.611 (6) Å	µ = 0.11 mm^−^^1^
*c* = 12.902 (9) Å	*T* = 296 K
β = 118.774 (8)°	Slab, colourless
*V* = 1221.3 (14) Å^3^	0.33 × 0.31 × 0.10 mm
*Z* = 2	

### Data collection

**Table:**

Bruker SMART CCD diffractometer	1745 reflections with *I* > 2σ(*I*)
Radiation source: fine-focus sealed tube	*R*_int_ = 0.037
graphite	θ_max_ = 26.0°, θ_min_ = 1.9°
ω scans	*h* = −15→15
12363 measured reflections	*k* = −10→10
2386 independent reflections	*l* = −15→15

### Refinement

**Table:**

Refinement on *F*^2^	Primary atom site location: structure-invariant direct methods
Least-squares matrix: full	Secondary atom site location: difference Fourier map
*R*[*F*^2^ > 2σ(*F*^2^)] = 0.047	Hydrogen site location: inferred from neighbouring sites
*wR*(*F*^2^) = 0.133	H atoms treated by a mixture of independent and constrained refinement
*S* = 1.06	*w* = 1/[σ^2^(*F*_o_^2^) + (0.0551*P*)^2^ + 0.5597*P*] where *P* = (*F*_o_^2^ + 2*F*_c_^2^)/3
2386 reflections	(Δ/σ)_max_ < 0.001
167 parameters	Δρ_max_ = 0.44 e Å^−^^3^
15 restraints	Δρ_min_ = −0.29 e Å^−^^3^

### Special details

**Table:**

Geometry. All e.s.d.'s (except the e.s.d. in the dihedral angle between two l.s. planes) are estimated using the full covariance matrix. The cell e.s.d.'s are taken into account individually in the estimation of e.s.d.'s in distances, angles and torsion angles; correlations between e.s.d.'s in cell parameters are only used when they are defined by crystal symmetry. An approximate (isotropic) treatment of cell e.s.d.'s is used for estimating e.s.d.'s involving l.s. planes.
Refinement. Refinement of *F*^2^ against ALL reflections. The weighted *R*-factor *wR* and goodness of fit *S* are based on *F*^2^, conventional *R*-factors *R* are based on *F*, with *F* set to zero for negative *F*^2^. The threshold expression of *F*^2^ > σ(*F*^2^) is used only for calculating *R*-factors(gt) *etc*. and is not relevant to the choice of reflections for refinement. *R*-factors based on *F*^2^ are statistically about twice as large as those based on *F*, and *R*- factors based on ALL data will be even larger.

### Fractional atomic coordinates and isotropic or equivalent isotropic displacement parameters (Å^2^)

**Table:**

	*x*	*y*	*z*	*U*_iso_*/*U*_eq_	
O1	0.2286 (2)	0.3041 (3)	−0.14930 (17)	0.0847 (7)	
O2	0.3228 (2)	0.5093 (3)	−0.16182 (17)	0.0896 (8)	
H2	0.2861	0.4876	−0.2324	0.134*	
O3	0.22033 (16)	0.5402 (2)	0.20189 (14)	0.0502 (5)	
O4	0.55406 (18)	0.2430 (2)	0.29251 (15)	0.0579 (5)	
O5	0.2066 (2)	1.0088 (2)	1.11020 (15)	0.0651 (6)	
C1	0.2949 (2)	0.4100 (3)	−0.1032 (2)	0.0487 (6)	
C2	0.3578 (3)	0.4407 (4)	0.0263 (2)	0.0575 (7)	
H2B	0.3407	0.5465	0.0395	0.069*	
H2C	0.4449	0.4321	0.0564	0.069*	
C3	0.3212 (3)	0.3327 (3)	0.09466 (19)	0.0479 (6)	
H3A	0.2332	0.3320	0.0597	0.057*	
H3B	0.3475	0.2281	0.0903	0.057*	
C4	0.3193 (2)	0.4830 (3)	0.26018 (19)	0.0388 (5)	
C5	0.4876 (2)	0.3319 (3)	0.30588 (19)	0.0403 (5)	
C6	0.5071 (2)	0.4122 (3)	0.41634 (19)	0.0366 (5)	
C7	0.6041 (2)	0.4039 (3)	0.5291 (2)	0.0396 (5)	
H7A	0.6714	0.3412	0.5482	0.048*	
C8	0.5939 (2)	0.4955 (3)	0.61186 (18)	0.0368 (5)	
C9	0.1607 (3)	0.8835 (4)	1.0674 (2)	0.0522 (7)	
H9A	0.170 (3)	0.794 (4)	1.119 (3)	0.066 (8)*	
C10	0.0679 (3)	0.9775 (4)	0.8676 (3)	0.0717 (9)	
H10A	0.1090	1.0710	0.9071	0.108*	
H10B	−0.0182	0.9958	0.8254	0.108*	
H10C	0.0955	0.9462	0.8131	0.108*	
C11	0.0398 (4)	0.7052 (5)	0.9101 (4)	0.1117 (16)	
H11A	0.0643	0.6347	0.9753	0.168*	
H11B	0.0663	0.6662	0.8566	0.168*	
H11C	−0.0472	0.7148	0.8695	0.168*	
N1	0.37472 (18)	0.3802 (2)	0.21817 (16)	0.0416 (5)	
N2	0.0942 (2)	0.8560 (3)	0.95392 (19)	0.0558 (6)	

### Atomic displacement parameters (Å^2^)

**Table:**

	*U*^11^	*U*^22^	*U*^33^	*U*^12^	*U*^13^	*U*^23^
O1	0.1149 (18)	0.0799 (15)	0.0394 (10)	−0.0332 (14)	0.0212 (11)	−0.0087 (10)
O2	0.1066 (17)	0.1158 (18)	0.0353 (10)	−0.0460 (15)	0.0254 (11)	0.0011 (11)
O3	0.0456 (10)	0.0599 (11)	0.0371 (9)	0.0041 (9)	0.0135 (8)	0.0081 (8)
O4	0.0728 (13)	0.0580 (11)	0.0467 (10)	0.0166 (10)	0.0319 (10)	−0.0008 (8)
O5	0.0889 (15)	0.0617 (12)	0.0383 (10)	−0.0119 (11)	0.0253 (10)	−0.0033 (9)
C1	0.0564 (14)	0.0563 (14)	0.0308 (11)	−0.0061 (12)	0.0190 (11)	−0.0015 (10)
C2	0.0695 (18)	0.0686 (18)	0.0319 (13)	−0.0228 (15)	0.0224 (13)	−0.0066 (12)
C3	0.0612 (16)	0.0484 (14)	0.0290 (12)	−0.0080 (12)	0.0178 (11)	−0.0050 (10)
C4	0.0435 (14)	0.0410 (13)	0.0314 (11)	−0.0031 (11)	0.0176 (11)	0.0046 (10)
C5	0.0514 (14)	0.0383 (12)	0.0341 (12)	0.0009 (11)	0.0228 (11)	0.0013 (9)
C6	0.0452 (13)	0.0361 (12)	0.0317 (11)	0.0017 (10)	0.0211 (10)	0.0030 (9)
C7	0.0409 (13)	0.0434 (13)	0.0352 (12)	0.0071 (10)	0.0188 (10)	0.0046 (10)
C8	0.0417 (13)	0.0408 (12)	0.0287 (11)	0.0005 (10)	0.0176 (10)	0.0044 (9)
C9	0.0573 (17)	0.0564 (17)	0.0404 (14)	0.0010 (13)	0.0215 (13)	0.0058 (13)
C10	0.0654 (19)	0.104 (3)	0.0398 (15)	0.0021 (18)	0.0207 (14)	0.0130 (15)
C11	0.110 (3)	0.078 (3)	0.088 (3)	−0.016 (2)	0.001 (2)	−0.017 (2)
N1	0.0513 (12)	0.0442 (11)	0.0278 (9)	−0.0014 (9)	0.0180 (9)	−0.0002 (8)
N2	0.0534 (13)	0.0621 (14)	0.0408 (12)	−0.0010 (11)	0.0139 (10)	−0.0024 (10)

### Geometric parameters (Å, °)

**Table:**

O1—C1	1.184 (3)	C6—C7	1.378 (3)
O2—C1	1.296 (3)	C6—C8^i^	1.387 (3)
O2—H2	0.8200	C7—C8	1.382 (3)
O3—C4	1.204 (3)	C7—H7A	0.9300
O4—C5	1.203 (3)	C8—C6^i^	1.387 (3)
O5—C9	1.222 (3)	C8—C4^i^	1.488 (3)
C1—C2	1.488 (3)	C9—N2	1.311 (3)
C2—C3	1.498 (4)	C9—H9A	0.99 (3)
C2—H2B	0.9700	C10—N2	1.445 (4)
C2—H2C	0.9700	C10—H10A	0.9600
C3—N1	1.458 (3)	C10—H10B	0.9600
C3—H3A	0.9700	C10—H10C	0.9600
C3—H3B	0.9700	C11—N2	1.449 (4)
C4—N1	1.387 (3)	C11—H11A	0.9600
C4—C8^i^	1.488 (3)	C11—H11B	0.9600
C5—N1	1.384 (3)	C11—H11C	0.9600
C5—C6	1.495 (3)		
			
C1—O2—H2	109.5	C6—C7—H7A	122.5
O1—C1—O2	122.5 (2)	C8—C7—H7A	122.5
O1—C1—C2	124.4 (2)	C7—C8—C6^i^	122.4 (2)
O2—C1—C2	113.1 (2)	C7—C8—C4^i^	129.6 (2)
C1—C2—C3	113.8 (2)	C6^i^—C8—C4^i^	107.94 (19)
C1—C2—H2B	108.8	O5—C9—N2	124.9 (3)
C3—C2—H2B	108.8	O5—C9—H9A	120.7 (17)
C1—C2—H2C	108.8	N2—C9—H9A	114.4 (18)
C3—C2—H2C	108.8	N2—C10—H10A	109.5
H2B—C2—H2C	107.7	N2—C10—H10B	109.5
N1—C3—C2	111.0 (2)	H10A—C10—H10B	109.5
N1—C3—H3A	109.4	N2—C10—H10C	109.5
C2—C3—H3A	109.4	H10A—C10—H10C	109.5
N1—C3—H3B	109.4	H10B—C10—H10C	109.5
C2—C3—H3B	109.4	N2—C11—H11A	109.5
H3A—C3—H3B	108.0	N2—C11—H11B	109.5
O3—C4—N1	125.2 (2)	H11A—C11—H11B	109.5
O3—C4—C8^i^	128.8 (2)	N2—C11—H11C	109.5
N1—C4—C8^i^	106.0 (2)	H11A—C11—H11C	109.5
O4—C5—N1	125.5 (2)	H11B—C11—H11C	109.5
O4—C5—C6	128.6 (2)	C5—N1—C4	112.35 (19)
N1—C5—C6	105.9 (2)	C5—N1—C3	124.1 (2)
C7—C6—C8^i^	122.5 (2)	C4—N1—C3	123.5 (2)
C7—C6—C5	129.7 (2)	C9—N2—C10	121.1 (3)
C8^i^—C6—C5	107.8 (2)	C9—N2—C11	121.6 (3)
C6—C7—C8	115.0 (2)	C10—N2—C11	117.3 (3)
			
O1—C1—C2—C3	−5.3 (4)	C6—C5—N1—C4	0.0 (3)
O2—C1—C2—C3	176.6 (3)	O4—C5—N1—C3	−1.9 (4)
C1—C2—C3—N1	−173.2 (2)	C6—C5—N1—C3	178.2 (2)
O4—C5—C6—C7	−0.5 (4)	O3—C4—N1—C5	−179.1 (2)
N1—C5—C6—C7	179.4 (2)	C8^i^—C4—N1—C5	0.6 (2)
O4—C5—C6—C8^i^	179.4 (2)	O3—C4—N1—C3	2.7 (4)
N1—C5—C6—C8^i^	−0.7 (2)	C8^i^—C4—N1—C3	−177.6 (2)
C8^i^—C6—C7—C8	−0.7 (4)	C2—C3—N1—C5	−88.7 (3)
C5—C6—C7—C8	179.2 (2)	C2—C3—N1—C4	89.2 (3)
C6—C7—C8—C6^i^	0.7 (4)	O5—C9—N2—C10	−0.8 (5)
C6—C7—C8—C4^i^	−179.0 (2)	O5—C9—N2—C11	−178.1 (4)
O4—C5—N1—C4	180.0 (2)		

Symmetry codes: (i) −*x*+1, −*y*+1, −*z*+1.

### Hydrogen-bond geometry (Å, °)

**Table:**

*D*—H···*A*	*D*—H	H···*A*	*D*···*A*	*D*—H···*A*
O2—H2···O5^ii^	0.82	1.78	2.583 (3)	166

Symmetry codes: (ii) *x*, −*y*+3/2, *z*−3/2.
